# Case Report: Decentralized trial of tolerability-adapted exercise therapy after severe Covid-19

**DOI:** 10.3389/fimmu.2025.1529385

**Published:** 2025-04-03

**Authors:** Jessica M. Scott, Zhuyu Qiu, Jahan Rahman, Chaya S. Moskowitz, Meghan G. Michalski, Sarah Lehman, Catherine P. Lee, Jenna Harrison, Anthony F. Yu, Amira Marouf, Santosha Vardhana, Paul C. Boutros, Lee W. Jones

**Affiliations:** ^1^ Department of Medicine, Memorial Sloan Kettering Cancer Center (MSK), New York, NY, United States; ^2^ Department of Medicine, Weill Cornell Medical College, New York, NY, United States; ^3^ Jonsson Comprehensive Cancer Center, University of California, Los Angeles, Los Angeles, CA, United States; ^4^ Department of Human Genetics, University of California, Los Angeles, Los Angeles, CA, United States; ^5^ Human Oncology and Pathogenesis Program, Memorial Sloan Kettering Cancer Center, New York, NY, United States; ^6^ Department of Medicine, Lymphoma Service, Memorial Sloan Kettering Cancer Center, New York, NY, United States; ^7^ Department of Urology, University of California, Los Angeles, CA, United States; ^8^ Institute for Precision Health, University of California, Los Angeles, CA, United States

**Keywords:** exercise, immune phenotype, long covid, cancer, decentralized

## Abstract

We assessed the safety, tolerability, and effects of exercise therapy in three patients with cancer and hospitalization for SARS-CoV-2 infection in an early-phase prospective trial. All study assessments and exercise sessions were conducted remotely (decentralized) in patient’s homes. Patients received five escalated doses of aerobic exercise therapy (range, 90 to 375 minutes per week) following a tolerability-based adapted schedule over 30 consecutive weeks. Exercise therapy was safe (i.e., no serious adverse events), tolerable (i.e., all exercise therapy doses were completed, with an overall average relative exercise dose intensity of 89%), and associated with improvements in patient physiology (e.g., exercise capacity) and patient-reported outcomes (e.g., quality of life). Correlative proteomic and single-cell immune sequencing of peripheral blood samples revealed marked alterations in protein and immune phenotypes implicated in post COVID-19 condition. (ClinicalTrials.gov number, NCT04824443).

## Introduction

According to the World Health Organization, there have been over 775 million cases of COVID-19 worldwide (1). Among these, an estimated 40% develop persistent symptoms lasting at least 2 months after initial severe acute respiratory syndrome coronavirus-2 (SARS-CoV-2) infection (2), a condition referred to as post COVID-19 condition (3). Post COVID-19 condition is characterized by multi-organ dysfunction and/or poor patient-reported outcomes (decreased overall quality of life (QOL), fatigue, exercise intolerance) (4–8). Cancer patients are particularly vulnerable to post COVID-19 condition, with reports of up to 60% prevalence (9). Dysregulation of immune processes is posited to underpin the severity and outcomes of post COVID-19 condition (10). At present, there are no approved strategies for the treatment or prevention of post COVID-19 condition (11).

Self-reported exercise is associated with lower risk of severe outcomes after SARS-CoV-2 infection including hospitalization and death (12, 13). Structured exercise therapy is a holistic strategy with potential to offset the multifaceted sequelae associated with post COVID-19 condition. The tolerability and benefits of exercise therapy after severe SARS-CoV-2 infection remains controversial, with concerns of post-exertional malaise exacerbation (14). To our knowledge, no study to date has evaluated the effects and correlative immune-inflammatory response to chronic exercise therapy in patients after severe SARS-CoV-2.

We conducted a prospective study of the feasibility, safety, and effects of tolerability-based aerobic exercise therapy in three cancer patients hospitalized for severe SARS-CoV-2 infection. This trial was conducted remotely, using a digital platform to perform all study procedures in patients’ homes (15). Correlative analyses included multimodal profiling of physiological response at high-frequency sampling with paired proteomic and single-cell immune sequencing of peripheral blood.

## Methods

### Patients and study design

This single-site, prospective non-randomized study enrolled three cancer patients with confirmed hospitalization for SARS-CoV-2 infection at Memorial Sloan Kettering Cancer Center (MSK) (NCT04824443). Patients were defined as non-exercising by 90 minutes of self-reported moderate- or higher-intensity exercise per week. Full methods are provided in the Supplement and study protocol. Briefly, pre-exercise screening included resting cardiac echocardiography to qualitatively assess systolic function, resting pulmonary function testing to assess forced expiratory volume in one second (FEV_1_), and an incremental, sub-maximal exercise tolerance test; all assessments were conducted in the patients’ home with real-time, remote guidance. Eligible patients potentially received five escalated exercise treatment doses: 90, 150, 225, 300, and 375 minutes per week following a dose adapted schedule. We used a dose-adapted schedule since our trial was the first to evaluate exercise therapy following severe SARS-CoV-2 infection and potential concerns of exercise therapy tolerability in this setting. Patients progressed through doses based on monitoring of exercise treatment tolerability (compliance) assessed by relative exercise dose intensity (REDI): the ratio of completed to planned dose for each exercise therapy session per patient (16). Mean REDI per patient was calculated over each distinct six-week treatment dose level evaluation period. A dose level was considered feasible if patients’ mean REDI was ≥70%, leading to dose-escalation.

Exercise therapy was administered across 3 to 6 individual treadmill walking sessions per week for 30 consecutive weeks or ~123 total planned unique treatment sessions. Sessions were conducted at patients’ individually-determined pre-treatment or mid-point exercise capacity according to a non-linear schedule (17). Sessions were performed remotely with real-time video monitoring by study exercise physiologists and continuous heart rate (Polar) and oxygen saturation (SpO_2_) (iHealth Air) measurement every 5 minutes.

All trial procedures were performed using the Digital Platform for Exercise (*DPEx*). *DPEx* involved remote *e*consent using video conferencing followed by shipment of an *e*tablet, treadmill, and several Bluetooth-enabled health devices to patients (15). Fasted blood samples were collected at pre- and post-treatment using a remote biospecimen collection service (Phlebotek, Inc).

### Lifestyle state, physiological and symptom assessment

Lifestyle (diurnal and nocturnal) state patterns were evaluated using a smartwatch (Withings ScanWatch). Changes in patient physiology were evaluated by: (1) exercise capacity was evaluated by a submaximal treadmill exercise tolerance test (time to 80% of age-predicted heart rate maximum) using a modified Balke-Ware protocol) at pre- and post-treatment, (2) body weight and body composition assessed daily using a wireless scale (Withings Body+), (3) resting heart rate using the smartwatch, (4) resting blood pressure assessed daily using a wireless blood pressure monitor (Withings BPM Connect), and (5) interstitial fluid glucose using continuous glucose monitoring (CGM; Abbott Freestyle Libre Pro system) at pre- and post-treatment. Patient reported outcomes (PRO) were evaluated electronically (ePROs) and included quality of life [Functional Assessment of Cancer Therapy–General] (18), and fatigue [Functional Assessment of Chronic Illness Therapy–Fatigue] (19) assessed at pre- and post-treatment.

### Peripheral blood proteomic and immune profiling

Detailed methods are provided in the Supplement. In brief, proteomics was conducted on plasma at pre- and post-treatment using SomaScan platform, a synthetic aptamer-based approach including a total of 7,596 different aptamers targeting distinct human protein epitopes (20). The assay used standard controls, including 12 hybridization normalization control sequences to control for variability in the Agilent readout process and 5 human calibrator control pooled replicates and 3 quality control pooled replicates to mitigate batch effects and verify the quality of the assay run using standard acceptance criteria. SomaScan assay data are first normalized using hybridization controls to mitigate variation within the run that comes from the readout steps: transfer to Agilent slides, hybridization, wash, and scan. This is followed by median signal normalization across pooled calibrator replicates within the run to mitigate within-run technical variation in the calibrator signal prior to use in scaling calculations. Profiling of peripheral blood mononuclear cells (PBMCs) was performed using the Single Cell Immune Profiling Platform (10x Genomics), as previously described (21). Single cell RNA FASTQ data was processed using Cellranger v7.0.0 *count* workflow to generate gene expression count matrices. TCR data was processed using Cellranger’s *vdj* pipeline to generate cell-clonotype annotations. Lane-wise demultiplexication was performed using Cite-seq-count v1.4.5. Processed RNA and ADT matrices were combined into a single *Seurat v4.0.0* object (22, 23) (using the *CreateSeuratObject* function), into which hashing oligo-sample assignment and TCR clonotype information was subsequently incorporated using scRepertoire v1.7.2 *combineExpression* function. Filtering was applied upon the merged data to only retain cells with (1) greater than 500 and less than 3000 detected unique RNA features, (2) less than 10000 total RNA molecules (3) less than 5 percent mitochondrial RNA reads and (4) less than 2500 total ADT molecules. RNA and ADT data were normalized using Seurat’s *NormalizeData* function with ‘LogNormalize’ and ‘CLR’ methods specified for each, respectively. PCA was performed (using Seurat’s *RunPCA* function) and to mitigate the effect of lane-specific and sample-specific covariates/batch effects, Harmony v1.0.0 *RunHarmony* function was used to correct embeddings (24). Elbow plots were manually inspected to determine the number of principal components to use downstream and a global UMAP was constructed using Seurat’s weighted nearest-neighbor workflow upon corrected RNA and ADT data. Using *clustree v.0.5.0* produced tree diagrams for various resolution input values, cluster stability was evaluated to avoid over-clustering and optimize the number of communities selected. Subsequently, separate Seurat object for Myeloid (monocytes and dendritic cells), B and T-lymphocytes were produced by evaluation of and isolation by canonical marker expression. The previously described UMAP construction and clustering steps were performed for each cell-subtype object. Specialized cell-types were labeled by manually evaluating differentially expressed genes and surface protein markers across clusters. Treatment-induced transcriptional and proteomic changes were characterized using Seurat’s implementation of the Wilcoxan-Rank Sum Test. *Gseapy v1.1.3* was used to evaluate-functional module level expression changes of various gene sets taken from the Molecular Signatures Database (MSigDB).

### Statistical analysis

Safety and tolerability of exercise therapy and changes in patient lifestyle and physiology were summarized using descriptive statistics. Gene Set Enrichment Analysis (GSEA) was conducted on plasma proteomic data at the patient level to evaluate changes from pre- to post-treatment. Transcriptional changes were characterized using *Seurat’s v4.0.0* implementation of the Wilcoxon-Rank Sum Test. *Fgsea v.1.24.0* and *escape v1.8.0* were used to evaluate-functional module level abundance changes of gene sets using the *Molecular Signatures Database (MSigDB).*

## Results

Patients were assessed for eligibility between August 2021 and March 2022. No pre-exercise therapy contraindications were identified, and all patients were asymptomatic at trial enrollment.

**Figure 1:**
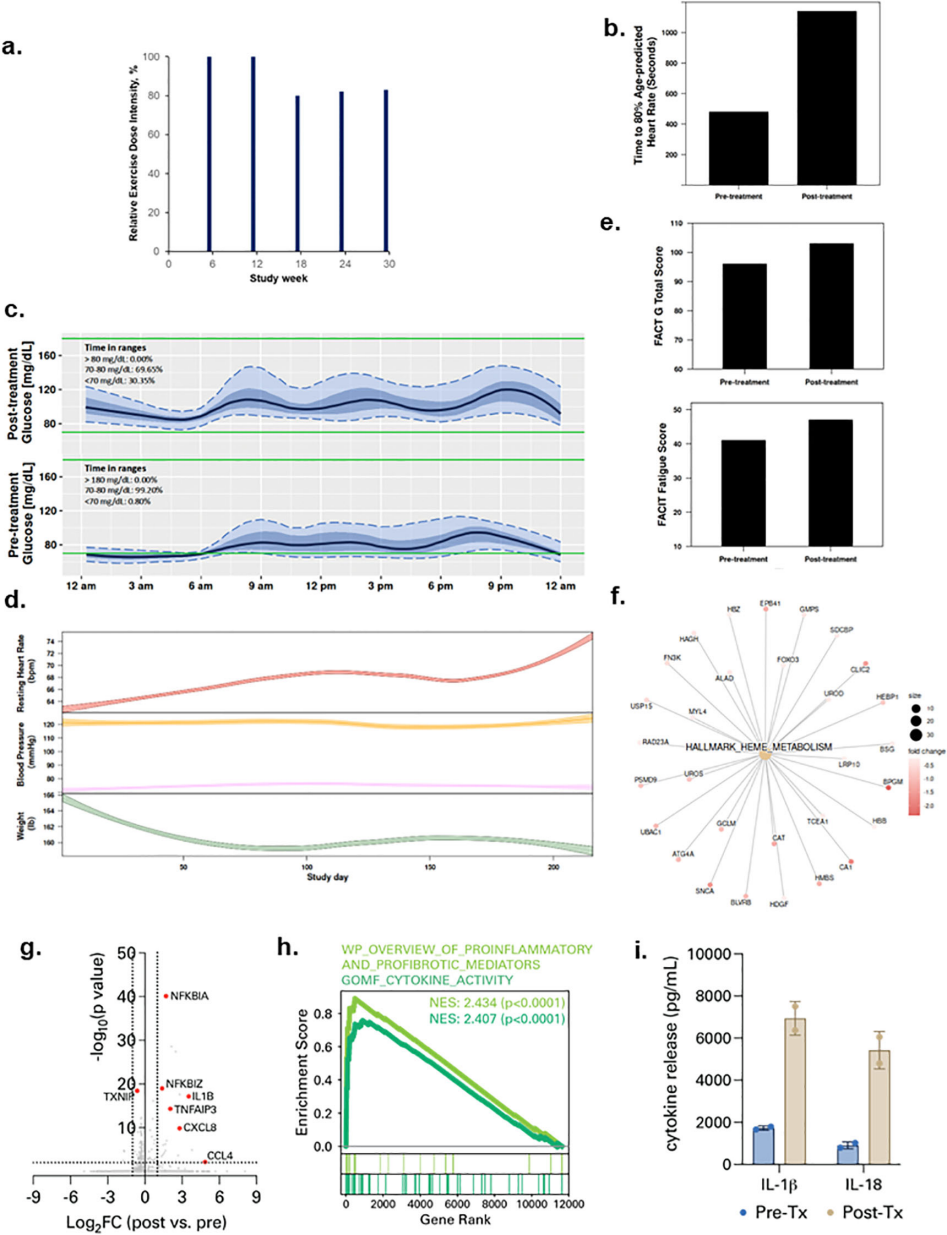
Tolerability and effects of aerobic exercise therapy following severe SARS-CoV-2 infection in Patient 1. **(a)** Patient 1 compliance to tolerability-adapted schedule as assessed by relative dose exercise intensity (ratio of planned to completed exercise therapy dose, expressed as a percentage). **(b)** changes in exercise capacity as measured by time to 80% of age-predicted heart rate from pre- to post-treatment. **(c)** differences in interstitial glucose levels (mg/dL) during sleep measured by time in pre-specified glucose ranges over a 24-hour period at pre-treatment (top panel) and post-treatment (bottom panel) Curves represent median (black line), 25^th^ and 75^th^ (interquartile range IQR, in darker blue), and 5^th^ and 95^th^ percentiles (in lighter blue) define the overall 24-h glucose profile. Green lines represent the targeted range: 70mg/dL - 180mg/dL). **(d)** time-series changes in resting heart rate (beats per minute) during sleep (top panel), resting systolic and diastolic blood pressure (mmHg) (middle panel), and body weight (Ibs) (bottom panel) over the study intervention. Data were smoothed using locally estimated scatterplot smoothing with colored band representing the 95% confidence interval. **(e)** changes in patient-reported overall quality of life (0-110) (top panel) and fatigue (0-50) (bottom panel; higher scores indicate lower fatigue) from pre-treatment to post-treatment. **(f)** enrichment map from Gene Set Enrichment Analysis on peripheral plasma proteomics at pre- and post-treatment. Dot size represent the fold change of the proteins corresponding to genes in enriched pathways. **(g)** differentially expressed genes in peripheral blood myeloid cells (PBMCs) post-treatment compared with pre-treatment. **(h)** enrichment of indicated cytokine-associated genesets. **(i)** spike protein-specific inflammasome activation in PBMCs post-treatment compared with pre-treatment.

Patient 1 was a 69-year-old male diagnosed with Stage 4 lymphoma in November 2018 and treated with R-Bendamustine. In January 2021 he was hospitalized for five days for SARS-CoV-2 infection, then re-admitted within 24 hours for an additional 13 days. At study enrollment in August 2021, his body mass index (BMI) was 28.5 kg/m^2^, with a predicted exercise capacity of 60% and FEV_1_ of 98%. All protocol-specified exercise therapy doses were completed with an overall REDI of 85% (range, 80 to 100% across doses, **Figure 1a**) (completed volume across doses: 90 mins/wk, 150 mins/wk, 180 mins/wk, 246 min/wk, 311 mins/wk). No serious adverse events (SAEs) were observed at any dose level; 3 non-serious AEs (n=2 tachycardia; n=1 arthralgia) occurred. No SpO_2_ desaturation events (SpO_2_ <90%) were observed during any exercise sessions. Lifestyle state profiling revealed stability for all states during study intervention (**Supplementary Figure S1**). Exercise capacity (time to 80% heart rate maximum) increased from 8:00 to 19:00 minutes (**Figure 1b**) while duration of interstitial fluid glucose lower glucose range (<70 mg/dl) increased from 0.8 to 30.3% from pre-treatment to post-treatment (**Figure 1c**). High-frequency sampling revealed reductions in body weight, increases in resting heart rate, and changes in blood pressure during study intervention (**Figure 1d**). QOL and fatigue improved by 7 and 6 points from pre-treatment to post-treatment, respectively (**Figure 1e**). Plasma proteomic analysis revealed downregulation of heme metabolism (**Figure 1f**), while single-cell immune profiling (**Supplementary Figures S2a-c**) showed an increase in circulating plasmacytoid dendritic cells (pDCs) (**Supplementary Figure S2d**) as well an increase in myeloid cell-specific NF-kB signaling and expression of chemokines such as *CCL4* (**Figures 1g, h**; **Supplementary Figure S2e**). Given that NF-kB signaling is a driver of inflammasome activation, we measured spike protein-driven inflammasome activation in M-CSF-differentiated macrophages from PBMCs (**Supplementary Figure S3**). Spike-driven inflammasome activity was potentiated after exercise treatment (**Figure 1i**; **Supplementary Figure S4**).

**Figure 2:**
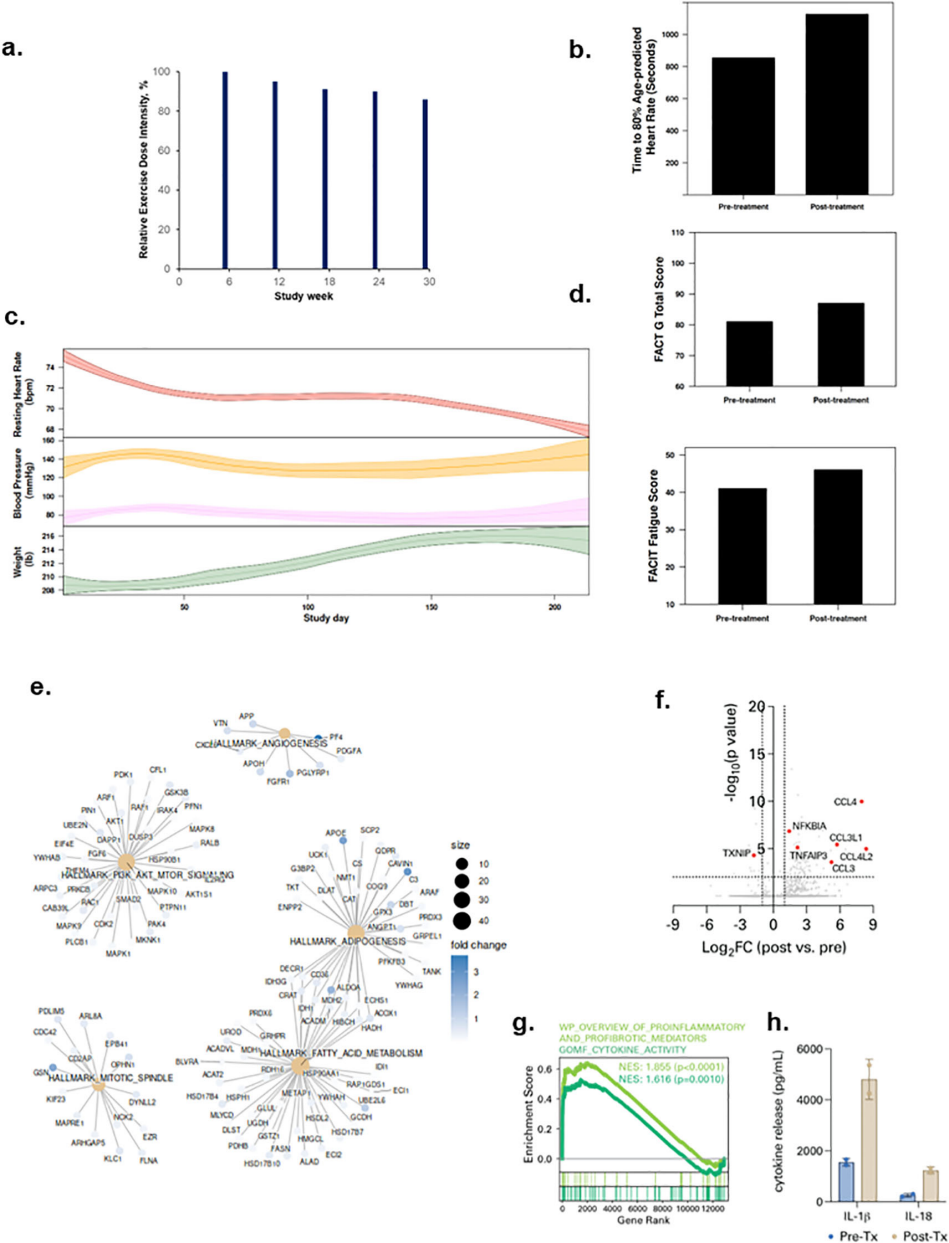
Tolerability and effects of aerobic exercise therapy following severe SARS-CoV-2 infection in Patient 2. **(a)** Patient 2 compliance to tolerability-adapted schedule as assessed by relative dose exercise intensity (ratio of planned to completed exercise therapy dose, expressed as a percentage). **(b)** changes in exercise capacity as measured by time to 80% of age-predicted heart rate from pre- to post-treatment. **(c)** time-series changes in resting heart rate (beats per minute) during sleep (top panel), resting systolic and diastolic blood pressure (mmHg) (middle panel), and body weight (Ibs) (bottom panel) over the study intervention. Data were smoothed using locally estimated scatterplot smoothing with colored band representing the 95% confidence interval. **(d)** changes in patient-reported overall quality of life (0-110) (top panel) and fatigue (0-50) (bottom panel; higher scores indicate lower fatigue) from pre-treatment to post-treatment. **(e)** enrichment map from Gene Set Enrichment Analysis on peripheral plasma proteomics at pre- and post-treatment. Dot size represent the fold change of the proteins corresponding to genes in enriched pathways. **(f)** differentially expressed genes in peripheral blood myeloid cells (PBMCs) post-treatment compared with pre-treatment. **(g)** enrichment of indicated cytokine-associated genesets. **(h)** spike protein-specific inflammasome activation in PBMCs post-treatment compared with pre-treatment.

Patient 2 was a 63-year-old male diagnosed with Stage 1 renal cell carcinoma in December 2020 and received a partial nephrectomy. In January 2021, he was hospitalized for SARS-CoV-2 infection for 7 days then discharged. At study enrollment in September 2021, his BMI was 30.3 kg/m^2^, predicted exercise capacity and FEV_1_ of 65% and 83%, respectively. He was receiving medication for hypertension, hyperlipidemia, and pulmonary embolism. All exercise therapy doses were completed with an overall REDI of 91% (range, 86 to 100% across dose escalations, **Figure 2a**) (completed volume across doses: 90 mins/wk, 143 mins/wk, 205 mins/wk, 270 min/wk, 323 mins/wk). No SAEs or SpO_2_ desaturations were observed; 7 non-serious AEs (n=5 tachycardia; n=2 backpain) were documented. Lifestyle state profiling revealed stability for all states except sedentary time which decreased during intervention (**Supplementary Figure S1**). From pre-treatment to post-treatment, exercise capacity increased from 14:15 mins to 18:47 mins (**Figure 2b**). During the study intervention, body weight increased while heart rate decreased (**Figure 2c**). QOL and fatigue improved by 6 and 5 points from pre-treatment to post-treatment, respectively (**Figure 2d**). Plasma proteomic analysis revealed enrichment of multiple gene sets involved in metabolism and angiogenesis (**Figure 2e**). Single-cell immune profiling showed a similar increase in pDCs (**Supplementary Figure S2d**), myeloid cell-specific NF-kB signaling, chemokine signaling, (**Figures 2f, g**; **Supplementary Figure S2e**) and spike protein-driven inflammasome activation (**Figure 2h**; **Supplementary Figure S4**) as observed in Patient 1.

**Figure 3:**
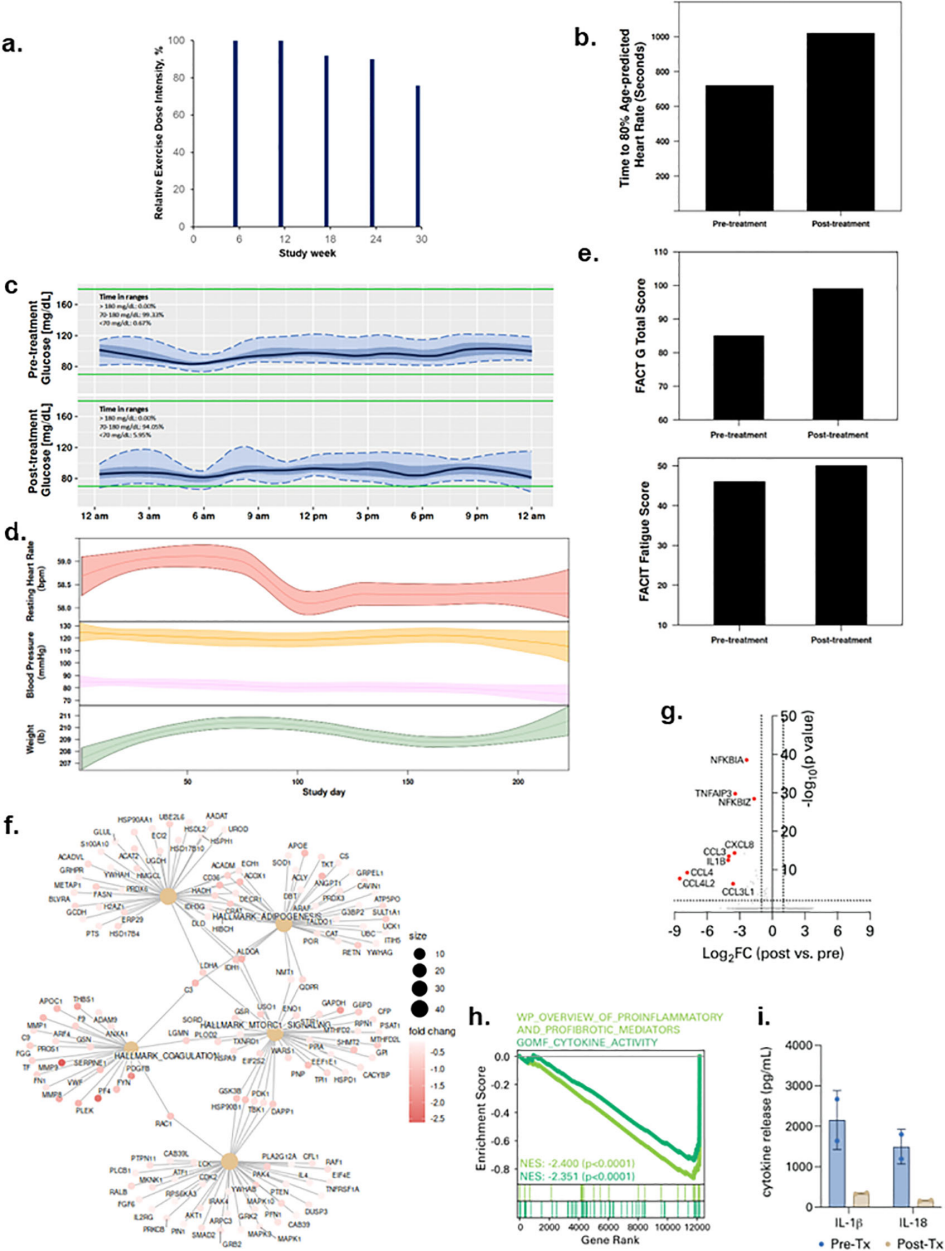
Tolerability and effects of aerobic exercise therapy following severe SARS-CoV-2 infection in Patient 3. **(a)** Patient 3 compliance to tolerability-adapted schedule as assessed by relative dose exercise intensity (ratio of planned to completed exercise therapy dose, expressed as a percentage). **(b)** changes in exercise capacity as measured by time to 80% of age-predicted heart rate from pre- to post-treatment. **(c)** differences in interstitial glucose levels (mg/dL) during sleep measured by time in pre-specified glucose ranges over a 24-hour period at pre-treatment (top panel) and post-treatment (bottom panel). Curves represent median (black line), 25^th^ and 75^th^ (interquartile range IQR, in darker blue), and 5^th^ and 95^th^ percentiles percentiles (in lighter blue) define the overall 24-h glucose profile. Green lines represent the targeted range: 70mg/dL - 180mg/dL). **(d)** time-series changes in resting heart rate (beats per minute) during sleep (top panel), resting systolic and diastolic blood pressure (mmHg) (middle panel), and body weight (Ibs) (bottom panel) over the study intervention. Data were smoothed using locally estimated scatterplot smoothing with colored band representing the 95% confidence interval. **(e)** changes in patient-reported overall quality of life (0-110) (top panel) and fatigue (0-50) (bottom panel; higher scores indicate lower fatigue) from pre-treatment to post-treatment. **(f)** enrichment map from Gene Set Enrichment Analysis on peripheral plasma proteomics at pre- and post-treatment. Dot size represent the fold change of the proteins corresponding to genes in enriched pathways. **(g)** differentially expressed genes in peripheral blood myeloid cells (PBMCs) post-treatment compared with pre-treatment. **(h)** enrichment of indicated cytokine-associated genesets. **(i)** spike protein-specific inflammasome activation in PBMCs post-treatment compared with pre-treatment.

Patient 3 was a 61-year-old male diagnosed with Stage 2 oral squamous cell carcinoma in August 2020 and treated with chemoradiation and high dose cisplatin. He was hospitalized for SARS-CoV-2 infection in December 2020 for 10 days then discharged. At trial enrollment (March 2022), he had a BMI was 28.5 kg/m^2^, with predicted exercise capacity and FEV_1_ of 79% and 82%, respectively. All exercise therapy dose levels were completed with an overall REDI of 90% (range, 76 to 100% across dose escalations, **Figure 3a**) (completed volume across doses: 90 mins/wk, 150 mins/wk, 207 mins/wk, 240 min/wk, 285 mins/wk). No SAEs or SpO_2_ desaturations were observed; 3 non-serious AEs (tachycardia, arthralgia, myalgia) occurred. All lifestyle states were stable except sedentary time which decreased during study intervention (**Supplementary Figure S1**). Exercise capacity increased from 12:00 mins to 17:00 mins (**Figure 3b**), time in lower interstitial fluid glucose (<70 mg/dl) increased from 0.6 to 5.9% from pre- to post-treatment (**Figure 3c**); decreases in heart rate and systolic and diastolic blood pressure were observed across study intervention (**Figure 3d**). QOL and fatigue improved by 14 and 4 points from pre- to post-treatment, respectively (**Figure 3e**). Plasma proteomic analysis revealed modulation of multiple gene sets, most notably downregulation of the complement system, inflammation, and coagulation pathways (**Figure 3f**). Consistent with this, parallel single-cell immune profiling showed striking downregulation in myeloid cell-specific NF-kB signaling (**Figures 3g, h**; **Supplementary Figure S2d**) and spike protein-driven inflammasome activation (**Figure 3i**; **Supplementary Figure S4**) following exercise therapy.

## Discussion

In this early phase prospective study, we explored the feasibility of monitored, personalized exercise therapy in adults after hospitalization for severe SARS-CoV-2 infection. Tolerability-based aerobic exercise therapy exercise therapy was safe, feasible, and associated with marked improvements in several physiologic measures as well as PROs. Effects on PROs are noteworthy since PROs were recommended as the primary end point for future clinical trials in post COVID-19 condition (11). Finally, the feasibility of conducting this trial remotely provides an attractive option for patients attempting to minimize exposures and/or unable to commute to site-based facilities due to disease limitations, public transit disruptions or other inconveniences. Intriguingly, two patients showed parallel increases in myeloid cell NF-kB and inflammasome activity. Active myeloid inflammation is associated with symptoms of post COVID-19 condition (25), but also may represent response to viral persistence (26). Although we cannot determine biological importance, this phenotype might confer either beneficial or pathological effects, depending on context. We speculate increased activity could indicate either: (1) an appropriate/successful response reflecting augmented myeloid cell-driven clearance of the COVID reservoir and improved homeostasis, or (2) an appropriate/unsuccessful response wherein pathogen clearance is not achieved, or (3) an inappropriate/unsuccessful response wherein sterile inflammation potentiates pathology. In contrast, Patient 3’s response was characterized by downregulation of blood protein and immune signatures present in patients with active post COVID-19 condition (27).

Limitations of our study require consideration. First, we conducted a “proof-of-concept” clinical trial; however, only data from randomized controlled trials can evaluate causality to determine whether the observed immune changes are due to exercise therapy or part of natural post-viral recovery. Second, proteomic and single-cell immune sequencing profiling were exploratory analyses to assess potential mechanisms underpinning effects of chronic exercise training and not part of standard of care management. Finally, our trial consisted of highly structured aerobic exercise therapy with individualized supervision. Exercise doses ranging from 90 to 375 min/wk were well-tolerated in all three patients; however, generalizability of our findings is restricted to post-curative intent therapy patients. Feasibility and safety of these doses could differ within other patient populations (e.g., patients with advanced disease). In conclusion, these initial findings suggest exercise therapy may regulate systemic immune-inflammatory processes implicated in post COVID-19 condition, with responses displaying inter-patient heterogeneity. Our hypothesis-generating findings may help guide future investigation of exercise therapy for prevention and/or management of post COVID-19 condition.

## Data Availability

The raw data supporting the conclusions of this article will be made available by the authors, without undue reservation.

## Supplementary material

Supplementary Figure 1 Time-series dynamic changes in lifestyle state across the study period for patients 1, 2, and 3. Data were smoothed using locally estimated scatterplot smoothing with colored band representing the 95% confidence interval.

Supplementary Figure 2 Uniform Manifold Approximation and Projection (UMAP) of **(a)** peripheral blood myeloid cells, and **(b)** myeloid/DC cells isolated from patients 1, 2 and 3 at pre-treatment (left panel) and post-treatment (right panel). **(c)** dot plot showing expression of genes defining myeloid cell subclusters shown in **(b)**. **(d)** relative abundance of myeloid cell subclusters at pre- and post-treatment. **(e)** changes in enrichment of indicated cytokine-associated genesets in myeloid cells post-treatment compared with pre-treatment, stratified by patient.

Supplementary Figure 3
**(a)** protocol for spike protein-dependent inflammasome activation in macrophages differentiated from the peripheral blood. **(b, c)** benchmarking of spike protein inflammasome activation shows nigericin-dependent inflammasome activation by both spike protein and LPS. **(d, e)** control experiments showing LPS and spike protein activation of TNF and IL-6 in the absence of nigericin.

Supplementary Figure 4
**(a)** spike protein-dependent inflammasome activation as measured by IL-1b or **(b)** IL-18 production in patients 1, 2, and 3, stratified by timepoint. Control peripheral blood myeloid cells treated without nigericin included as a negative control.
