# Supplementary material for: Case Report: Decentralized trial of tolerability-adapted exercise therapy after severe Covid-19
Source: Front Immunol. 2025 Apr 3;16:1529385. doi: 10.3389/fimmu.2025.1529385 (PMC12003135; doi:10.3389/fimmu.2025.1529385)
Supplement: Supplementary file 1 [file DataSheet1.pdf]

Supplemental Methods

Case Report: Decentralized trial of tolerability-adapted exercise therapy after severe Covid-19

Supplemental methods

Patients and setting. This was a single center, proof-of-concept study of exercise therapy in patients with cancer and confirmed hospitalization for SARS-CoV-2 infection at Memorial Sloan Kettering Cancer Center (MSK) (NCT04824443). Other major eligibility were: (1) aged ≥18 years, (2) discharged from hospital for at least 30 days, (3) Interval of ≥12 months but ≤10 years following completion of primary non-hormonal anti-cancer therapy (current endocrine therapy and maintenance therapy allowed), (4) space to house a treadmill, and (5) cleared for participation in home-based moderate-intensity exercise therapy as per screening clearance.

Major exclusion criteria were: (1) receiving home oxygen after discharge, (2) admitted to critical care for SARS-CoV-2 infection, (3) enrollment onto any other interventional investigational study, (4) presence of any other concurrent, actively treated malignancy, (5) distant metastatic malignancy, (6) receiving non-hormonal anti-cancer therapy, and (7) any other condition or intercurrent illness that, in the opinion of the investigator, makes the subject a poor candidate for study participation. The study was approved by the Memorial Sloan Kettering (MSK) Cancer Center institutional review board. All patients provided written informed consent. Study enrollment was conducted at MSK between August 2021 and March 2022.

Study procedures. Potential patients were identified using DataLine. DataLine uses structured queries and natural language processing to search the institution's database warehouse (integration of all clinical and operational subject areas) to screen on trial-specific eligibility criteria: type of cancer diagnosis, visit type, geographical distance from MSK, primary language, and provider. The generated list was then cross-referenced against MSK's scheduling system to confirm surgery type and date. The primary MD provider was then contacted by email to confirm eligibility and permission for patient contact.

Potential eligible patients were contacted by study staff. Interested patients were provided with a detailed overview of study procedures. In those remaining interested, study staff confirmed other major eligibility criteria (*e.g.,* sufficient space for in-home treadmill) and if all eligibility criteria were met, a convenient date and time was scheduled to conduct the informed consent discussion. Informed consent was performed using a video conferencing platform. Specifically, patients (located at their preferred location) received a secure link via the MyMSK patient portal which connected them to the video call; a member of the study team then shared their screen following verification of standard patient identification (spelling of first and last name, date of birth and/or MRN). The patient and study staff member were able to review the MSK *e*Consent module (MSK eConsent System, v2) simultaneously in real-time. Consenting patients were provided remote screen control or sent a link to sign the informed consent, a copy was then sent to the patient *via* mail, email, or Portal Secure Messaging (PSM) depending on preference.

Following final verification of eligibility and "written" informed consent, patients were couriered a study kit containing an etablet and multiple Bluetooth-enabled devices including activity and heart rate monitor (Withings Steel HR), blood pressure monitor (Withings BPM Connect), a body composition scale (Withings Body+), oxygen saturation (SpO_2) monitor (iHealth Air) and continuous glucose monitor. These devices were connected to a heath data platform (Validic Inc.), a cloud-based, HIPPA-compliant technology, securely connecting passively collected, deidentified data to MSK's patient portal system. Splunk (v7.2.7) was utilized for data storage and analytics using a custom dashboard for monitoring of connected device data. Patients were also provided with a treadmill (Jog Forma or MyRun, Technogym, Inc), installation performed by Technogym, Inc. technicians. Next, study staff conducted a two-way video orientation session to overview use of all study devices, study timeline and expectations, and implement a submaximal exercise tolerance test. This test was performed in the patients' home, remotely administered, and supervised using video conferencing. Following baseline assessments, study staff arranged a convenient time for a mobile phlebotomy service (Phlebotek Solutions Inc.) to perform in-home blood collection (patients could also choose to undergo blood draw procedures at a MSK facility) and scheduled the first remote exercise therapy session. After study completion, all study devices were returned to MSK using a pre-paid courier service whereas the treadmill was extracted, serviced, and stored by Technogym, Inc. for future use.

Exercise therapy. Exercise therapy was initiated within seven days of informed consent. Exercise therapy was standardized between patients based on modality, dose intensity, progression, and schedule. Dedicated study

personnel with at least bachelor's degrees in Exercise Science implemented the interventions and individually monitored all sessions. Exercise physiologists' adherence to the protocol was reviewed on weekly basis by the Principal Investigator (LWJ). All sessions were by appointment only using MSK's electronic scheduling system (Epic Hyperspace), with patients contacted within 24 hours of a scheduled missed session. Rescheduling of missed sessions was permitted within each exercise dosing level.

Patients potentially received five escalated exercise treatment doses: (i) 90 minutes per week: 30 minutes per treatment, 3 times weekly, (ii) 150 minutes per week: ~30 minutes per treatment five times weekly, (ii) 225 minutes per week: ~45 minutes per treatment five times weekly, (iv) 300 minutes per week: ~60 minutes per treatment, 5 times weekly, (v) 375 minutes per week: ~60 minutes per treatment, 6 times weekly following a dose adapted schedule. Patients progressed through doses based on monitoring of exercise treatment tolerability (compliance) assessed by relative exercise dose intensity (REDI): the ratio of completed to planned dose for each exercise therapy session per patient.[1] Mean REDI per patient was calculated over each distinct six-week treatment dose level evaluation period. A dose level was considered feasible if patients' mean REDI was ≥70%, leading to dose-escalation.

After a two week "ramp-up" period in which duration and/or intensity were progressively increased, all subsequent sessions starting in week two within each dose level were conducted using four different intensities (*i.e.,* 50-60%, 65-70%, 70-75%, and 80-85%) of measured baseline or midpoint exercise capacity. The intensity was tailored to each patient based on the workload (*i.e.,* treadmill speed/grade) corresponding to a specific percentage of workload measured during the exercise capacity test. The duration of individual sessions ranged from 20 minutes to 75 minutes.

Sessions were performed in the patients' residence with remote supervision and monitoring by study personnel. If supervised sessions were not possible, patients were able to perform a session without supervision, but such sessions could only be those assigned at lower intensity (<65% of exercise capacity).

Prior to the initiation of all sessions, the patient measured resting blood pressure, heart rate, and SpO_2 using the devices provided in the study kit. Patients were also queried whether they adhered to MSK Exercise-Oncology's pre-exercise guidelines.[2,3] The patient then verbally communicated the blood pressure and heart rate readings to the attending exercise physiologist supervising the session. The planned session was only initiated if vital signs were within acceptable limits according to MSK Exercise-Oncology's guidelines.[2] In addition, the planned session was also not initiated if the exercise physiologist observed any concerns that may compromise participant safety and/or the integrity of the planned session. Next, participants were instructed to enter an individualized speed (mph) and incline (%), based on their planned training zone, into the treadmill to initiate warm-up (~5 minutes), following by the planned treatment session. During each session, the exercise physiologist recorded exercise therapy dose metrics (duration, speed, incline, heart rate, rating of perceived exertion), as well as any dose modifications, in real-time *via* clinical electronic recording forms synced with each patients' electronic health record (EHR).

Safety and verification of all sessions was evaluated using continuous monitoring of heart rate recorded on electronic recording forms linked to the EHR. Toxicity grading was performed in accordance with National Cancer Institute's Common Terminology Criteria for Adverse Events v5.0. Adverse events (AEs) were reviewed by the attending exercise physiologist, then graded and attributed following exercise sessions. AEs not resolved at the end of a session were reviewed with the patient at the beginning of the next scheduled session. During the intervention phase, AEs requiring adjustments to the planned prescription were performed according to the MSK Exercise-Oncology standards of care and/or at the discretion of the Principal Investigator (LWJ).

All data was automatically synced daily into the institutional database for storage, and linked to Tableau for visualization, permitting staff to overview exercise-related data (compliance, events) in real-time. Dose modification was permitted and performed using standardized criteria. The planned dose of all sessions was quantified as metabolic equivalent task (MET)– hours per session. The planned intensity of each session was multiplied by the corresponding session duration to calculate METs per session; all sessions were summed to derive total planned cumulative MET-hours per patient (*i.e.,* relative exercise dose intensity, REDI).[1]

Assessments

Pre-exercise cardiovascular screening

Systolic function was evaluated using tele-guided self-ultrasound scanning.[4] Participants were shipped an ultrasound probe (Lumify, Philips) and ultrasound gel, and were remotely guided to obtain cardiac images at baseline. Images were qualitatively evaluated by a cardiologist to confirm adequate systolic function.

Pulmonary function was evaluated using a tele-guided pulmonary function test at baseline, midpoint, and follow-up. Participants were shipped a spirometer (GoSpiro, MTI), and were remotely guided through a pulmonary function test to evaluate forced expiratory volume in one second and forced vital capacity.[5]

Safety was evaluated by the type and prevalence of serious (*e.g.,* life-threatening, hospitalization, significant incapacity, important medical events) and non-serious (*e.g.,* knee, back pain) adverse events during exercise therapy sessions. Exercise therapy session heart rate and blood pressure response was compared to vitals obtained during the exercise tolerance test. Only patients with physician clearance were able to proceed with participation. AEs were reviewed by exercise physiologists and graded and attributed at the end of each session. AEs were counted once per patient as one entity.

Tolerability was assessed by multiple endpoints including: rate of lost to follow up (*i.e.,* completion of postintervention assessments), attendance (ratio of total attended to planned treatments), permanent discontinuation (treatment discontinuation prior to postintervention assessments), treatment interruption (missing ≥ 3 consecutive planned sessions), dose modification [≥ 10% of sessions requiring modification (reduction / escalation) of intensity and/or duration], pre-treatment dose modification (reduction of pre-treatment session intensity), early session termination (termination of session prior to planned duration), and REDI.[3]

Lifestyle states and physiological response was assessed by evaluating change in: (1) diurnal and nocturnal patterns [i.e., sleep, sedentary, mobility (non-exercise)] evaluated by a smartwatch with infrared (wrist) sensor;
(2) exercise capacity evaluated by a submaximal exercise tolerance test. Briefly, participants will begin walking on treadmill at a participant-specific designated speed and 0% incline. A combination of staged increases to speed and/or incline will occur throughout the test until patients reach 80% of their age-predicted heart rate maximum ((((220 - Age) - Resting HR) x 0.80) + Resting HR) or symptom limitation. The test was performed in the patients' residence using the study treadmill with remote real-time supervision and monitoring by staff exercise physiologists using a video conferencing platform. All assessment-related data was recorded using electronic recording forms synced with the EHR. Exercise tolerance testing was performed at baseline and repeated within seven days of the final intervention session at post-treatment. Age-predicted values were calculated using the Fitness Registry and the Importance of Exercise National Database equation.[6]
(3) anthropometrics (*i.e.,* body weight, body composition) evaluated daily using a wireless scale;
(4) heart rate and resting blood pressure were evaluated every 10 minutes 24/7 using the wireless smartwatch (Withings Steel HR) and daily using a wireless blood pressure monitor, respectively,
and;
(5) continuous interstitial fluid glucose monitoring (CGM). CGM was performed using the Abbott Freestyle Libre Pro system, providing measurement of interstitial glucose concentrations every 15 minutes for up to 14 days. Specifically, the CGM kit included a prepackaged sensor, applicator, and reader to activate the sensor. After 14 days of continual wear, the sensor was removed and returned to MSK study staff for analysis using a pre-paid courier service. The Abbott Freestyle Libre Pro system sensor does not meet the FDA Significant Risk definition under 21 CFR 812.3(m) and therefore was determined to be a Non-Significant Risk device.

Patient-reported symptoms comprised of: (1) quality of life [Functional Assessment of Cancer Therapy–General],[7] and fatigue [Functional Assessment of Chronic Illness Therapy–Fatigue][8] were assessed at pre- and post-treatment.

Peripheral blood samples at pre- and post-treatment were collected either *via* a remote (in-home) blood collection service or via standard of care clinical visits at a MSK facility. Remote (in-home) blood collection was coordinated using Workpath, Inc. Workpath – a HIPAA compliant platform – organizes, verifies, and dispatches mobile medical services. Using the Workpath dashboard and partnering with Phlebotek, Inc. a third-party mobile phlebotomy vendor, appointments were scheduled for a trained phlebotomist to visit a patients' residence to collect fasted blood samples. The dashboard also permits real-time monitoring enabling blood collection and

delivery to be tracked remotely by the Exercise Oncology study team. Participants were instructed to adhere to Exercise-Oncology blood collection procedures prior to every blood collection. Blood collection was scheduled between 0700 and 1000 h, after an 8-hr water-only fast. Samples were then transported to a designated MSK facility for processing and stored at −80 °C.

Plasma proteomics. The SomaScan Platform for proteomic profiling uses 4979 SOMAmer reagents, single-stranded DNA aptamers, to 4776 unique human protein targets. The modified aptamer binding reagents, SomaScan assay, its performance characteristics, and specificity to human targets have been previously described. The assay used standard controls, including 12 hybridization normalization control sequences to control for variability in the Agilent readout process and 5 human calibrator control pooled replicates and 3 quality control pooled replicates to mitigate batch effects and verify the quality of the assay run using standard acceptance criteria. SomaScan assay data are first normalized using hybridization controls to mitigate variation within the run that comes from the readout steps: transfer to Agilent slides, hybridization, wash, and scan. This is followed by median signal normalization across pooled calibrator replicates within the run to mitigate within-run technical variation in the calibrator signal prior to use in scaling calculations. The set of ratios of the calibrator reference value to the median of calibrator replicates for each SOMAmer® reagent is calculated and decomposed into two terms: plate scale - the median ratio, and calibration scale - the recalculated set of scale factors, one for each SOMAmer reagent. Plate scale adjusts for overall signal intensity differences between runs. Calibration adjusts for SOMAmer reagent-specific assay differences between runs. Median signal normalization is performed using Adaptive Normalization by Maximum Likelihood (ANML) for specimen types and studies shown to be consistent with predefined population references or, alternatively, using median normalization to a study specific reference.

Peripheral blood immune cell profiling. Single cell RNA FASTQ data was processed using Cellranger v7.0.0 *count* workflow to generate gene expression count matrices. TCR data was processed using Cellranger's *vdj* pipeline to generate cell-clonotype annotations. Lane-wise demultiplexication was performed using Cite-seq-count v1.4.5. Processed RNA and ADT matrices were combined into a single *Seurat v4.0.0* object[9,10] (using the *CreateSeuratObject* function), into which hashing oligo-sample assignment and TCR clonotype information was subsequently incorporated using scRepertoire v1.7.2 *combineExpression* function. Filtering was applied upon the merged data to only retain cells with (1) greater than 500 and less than 3000 detected unique RNA features, (2) less than 10000 total RNA molecules (3) less than 5 percent mitochondrial RNA reads and (4) less than 2500 total ADT molecules. RNA and ADT data were normalized using Seurat's *NormalizeData* function with 'LogNormalize' and 'CLR' methods specified for each, respectively.

PCA was performed (using Seurat's *RunPCA* function) and to mitigate the effect of lane-specific and sample-specific covariates/batch effects, Harmony v1.0.0 *RunHarmony* function was used to correct embeddings[11]. Elbow plots were manually inspected to determine the number of principal components to use downstream and a global UMAP was constructed using Seurat's weighted nearest-neighbor workflow upon corrected RNA and ADT data. Using *clustree v.0.5.0* produced tree diagrams for various resolution input values, cluster stability was evaluated to avoid over-clustering and optimize the number of communities selected. Subsequently, separate Seurat object for Myeloid (monocytes and dendritic cells), B and T-lymphocytes were produced by evaluation of and isolation by canonical marker expression. The previously described UMAP construction and clustering steps were performed for each cell-subtype object. Specialized cell-types were labeled by manually evaluating differentially expressed genes and surface protein markers across clusters. Treatment-induced transcriptional and proteomic changes were characterized using Seurat's implementation of the Wilcoxan-Rank Sum Test. *Gseapy v1.1.3* was used to evaluate-functional module level expression changes of various gene sets taken from the Molecular Signatures Database (MSigDB).

Spike protein-driven inflammasome activation. Inflammasome activation was performed as previously described[12]. Briefly, CD14+ cells were isolated from PBMCs by positive selection using a monocyte isolation kit (Miltenyi Biotch, Bergisch Gladbach, Germany. 5 × 10^4 CD14+ cells were cultured for 5 days in Roswell Park Memorial Institute (RPMI) 1640 Medium (Thermo Fisher Scientific, Waltham, MA, USA) containing 10% fetal bovine serum (Thermo Fisher Scientific) and 50 ng/ml M-CSF (Miltenyi Biotec) to promote macrophage

differentiation. Differentiated macrophages were stimulated with/without recombinant SARS-CoV-2 spike protein (0.1 μg/ml) or lipopolysaccharide (LPS) for 4 hours. To activate IL-1β secretion nigericin or ATP was added for 2 hours, after which supernatants were harvested. IL-1β secretion in the culture supernatant was quantified by ELISA.

Statistical considerations and data analysis. Safety, tolerability, and changes in patient lifestyle, physiology, and quality of life were analyzed using descriptive statistics. Time series data for individual patient physiological measures and lifestyle states were visualized with line plots using locally estimated scatterplot smoothing (LOESS) and 95% confidence intervals. CGM data were summarized and collapsed into a single 24 hour "modal day", presenting the 5th, 25th, 50th, 75th, and 95th percentiles using the R package *iglu*(v4.1.6). Patient lifestyle patterns were categorized into five states—sleep, sedentary, active (non-exercise), intermittent, and exercise—at a per-minute resolution across the study period and visualized similarly with LOESS-smoothed line plots and 95% confidence intervals.

For proteomic analyses of peripheral blood samples, comparisons between baseline and post-intervention samples were conducted using Gene Set Enrichment Analysis (GSEA) with the R package *clusterProfiler* (v4.10.1). Data analyses were performed in the R statistical environment (v3.4.1). Data visualizations were conducted jointly using the *Boutros.Lab.plotting.general* (v7.1.0), *ggplot2* (v3.5.1), *enrichplot* (v1.22.0), and *iglu* (v4.1.6) packages.

For single-cell analysis of peripheral blood samples, treatment-induced transcriptional and proteomic changes were characterized using Seurat's implementation of the Wilcoxon-Rank Sum Test. *Fgsea* (v.1.24.0) and *escape* (v1.8.0) were used to evaluate-functional module level expression changes of various gene sets taken from the Molecular Signatures Database (MSigDB).

For spike protein-induced inflammasome activation, experiments were performed for each patient in biological duplicates. Statistical analysis was performed using a student's t-test.

Study oversight. The trial was designed and conducted by the first and senior author. All the authors confirm that the trial conformed to the protocol and attest to the accuracy and completeness of the data. The senior author wrote the first draft of the manuscript. The first and senior authors had full access to the data. All authors were involved in data interpretation, in writing and reviewing subsequent manuscript drafts, and in making the decision to submit the manuscript for publication. Trial conduct was monitored by a Data Safety and Monitoring Board.

Data sharing. Qualified researchers can request access to deidentified participant data that underlie this Article through submission of a proposal with a valuable research question to the corresponding author, provided that the necessary data protection agency and ethical committee approvals follow the relevant registration. A contract will also be signed.

Supplemental References

1. Nilsen TS, Scott JM, Michalski M, et al. Novel Methods for Reporting of Exercise Dose and Adherence: An Exploratory Analysis. Med Sci Sports Exerc 2018;50(6):1134-1141. DOI: 10.1249/MSS.0000000000001545.
2. Scott JM, Thomas SM, Peppercorn JM, et al. Effects of Exercise Therapy Dosing Schedule on Impaired Cardiorespiratory Fitness in Patients With Primary Breast Cancer: A Randomized Controlled Trial. Circulation 2020;141(7):560-570. DOI: 10.1161/CIRCULATIONAHA.119.043483.
3. Scott JM, Iyengar NM, Nilsen TS, et al. Feasibility, safety, and efficacy of aerobic training in pretreated patients with metastatic breast cancer: A randomized controlled trial. Cancer 2018;124(12):2552-2560. DOI: 10.1002/cncr.31368.
4. Scott JM, Downs M, Martin DS, et al. Teleguided self-ultrasound scanning for longitudinal monitoring of muscle mass during spaceflight. iScience 2021;24(4):102344. DOI: 10.1016/j.isci.2021.102344.
5. Kreider ME, Grippi MA. Impact of the new ATS/ERS pulmonary function test interpretation guidelines. Respir Med 2007;101(11):2336-42.
6. Kokkinos P, Kaminsky LA, Arena R, Zhang J, Myers J. New Generalized Equation for Predicting Maximal Oxygen Uptake (from the Fitness Registry and the Importance of Exercise National Database). Am J Cardiol 2017;120(4):688-692. DOI: 10.1016/j.amjcard.2017.05.037.
7. Cella DF, Tulsky DS, Gray G, et al. The Functional Assessment of Cancer Therapy scale: development and validation of the general measure. J Clin Oncol 1993;11(3):570-9. (Research Support, Non-U.S. Gov't
Research Support, U.S. Gov't, P.H.S.) (In eng) (http://www.ncbi.nlm.nih.gov/pubmed/8445433).
8. Cella D. The Functional Assessment of Cancer Therapy-Anemia (FACT-An) Scale: a new tool for the assessment of outcomes in cancer anemia and fatigue. Semin Hematol 1997;34(3 Suppl 2):13-9.
9. Hao Y, Hao S, Andersen-Nissen E, et al. Integrated analysis of multimodal single-cell data. Cell 2021;184(13):3573-3587 e29. DOI: 10.1016/j.cell.2021.04.048.
10. Stuart T, Butler A, Hoffman P, et al. Comprehensive Integration of Single-Cell Data. Cell 2019;177(7):1888-1902 e21. DOI: 10.1016/j.cell.2019.05.031.
11. Korsunsky I, Millard N, Fan J, et al. Fast, sensitive and accurate integration of single-cell data with Harmony. Nat Methods 2019;16(12):1289-1296. DOI: 10.1038/s41592-019-0619-0.
12. Theobald SJ, Simonis A, Georgomanolis T, et al. Long-lived macrophage reprogramming drives spike protein-mediated inflammasome activation in COVID-19. EMBO Mol Med 2021;13(8):e14150. DOI: 10.15252/emmm.202114150.
