# Supplementary material for: Case Report: Decentralized trial of tolerability-adapted exercise therapy after severe Covid-19
Source: Front Immunol. 2025 Apr 3;16:1529385. doi: 10.3389/fimmu.2025.1529385 (PMC12003135; doi:10.3389/fimmu.2025.1529385)

Supplemental Figure 1.

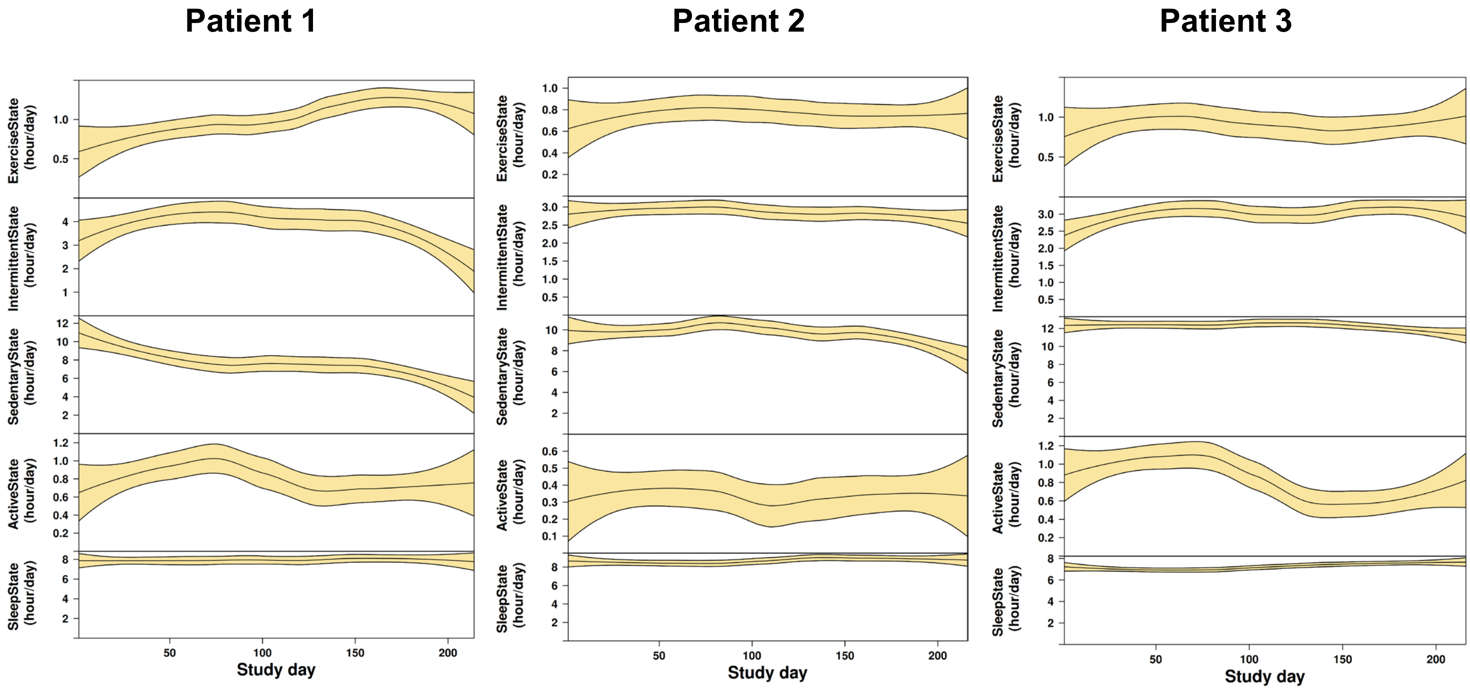

Supplemental Figure 2.

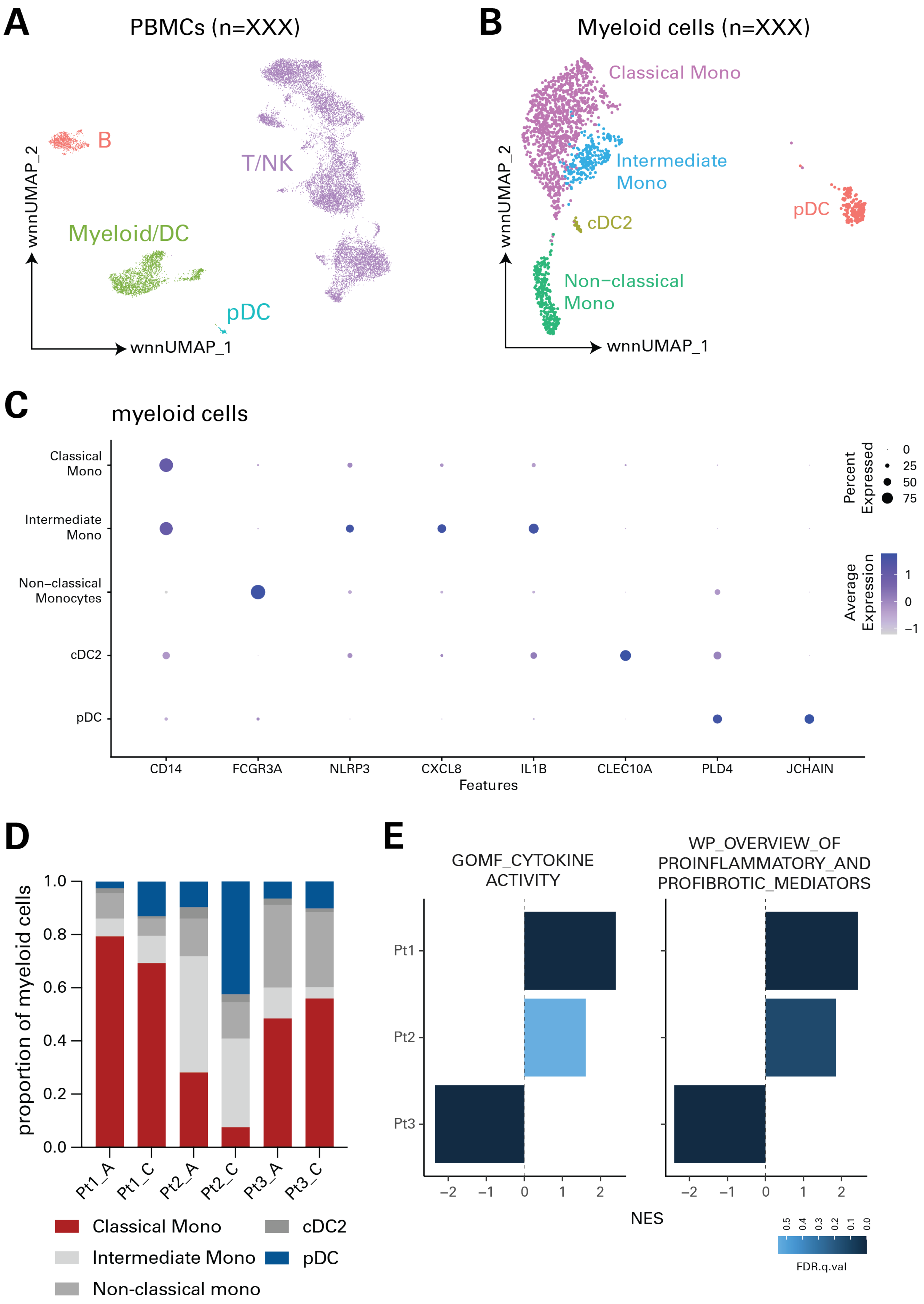

Supplemental Figure 3.

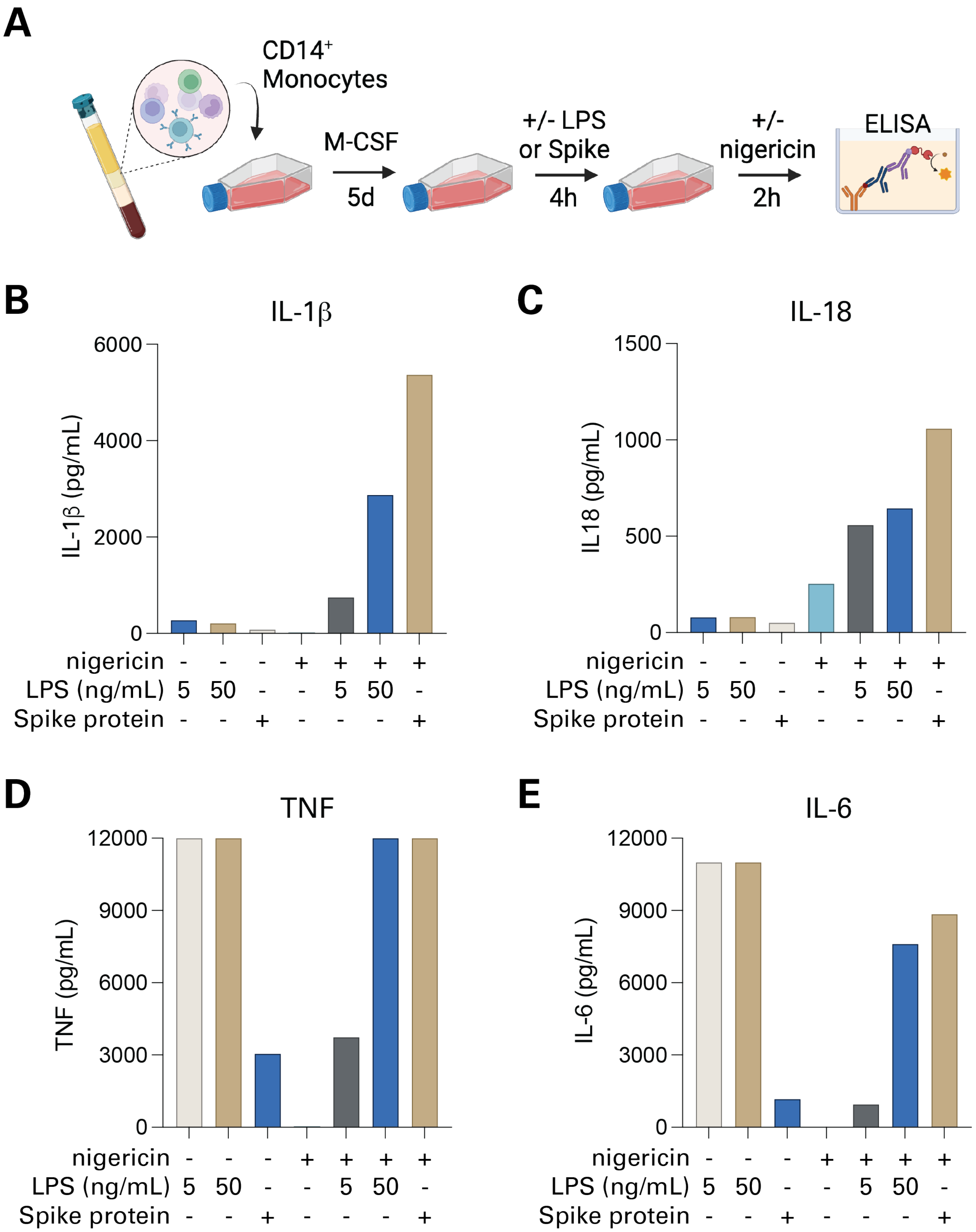

Supplemental Figure 4.

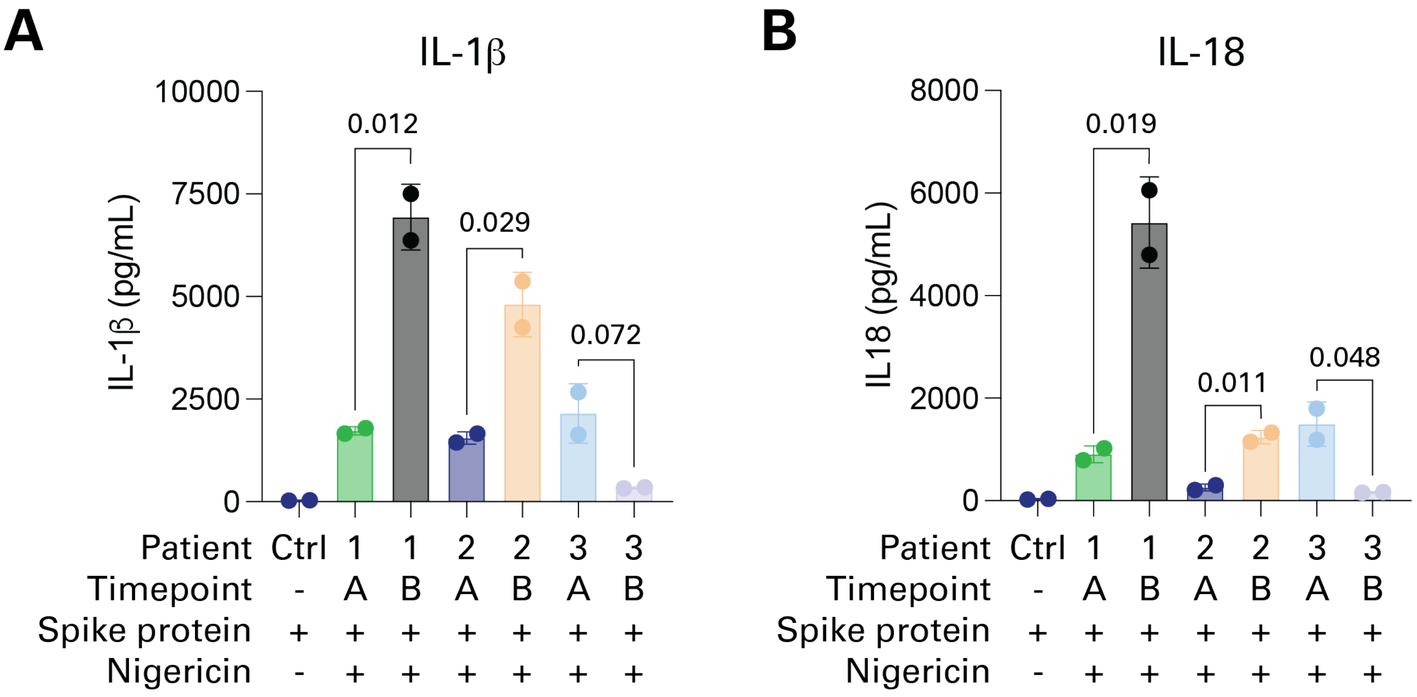

Supplement: Supplementary Figure 1 — Time-series dynamic changes in lifestyle state across the study period for patients 1, 2, and 3. Data were smoothed using locally estimated scatterplot smoothing with colored band representing the 95% confidence interval. [file DataSheet2.pdf]
